# Refuting the myth of a ‘tsunami’ of mental ill-health in populations affected by COVID-19: evidence that response to the pandemic is heterogeneous, not homogeneous

**DOI:** 10.1017/S0033291721001665

**Published:** 2021-04-20

**Authors:** Mark Shevlin, Sarah Butter, Orla McBride, Jamie Murphy, Jilly Gibson-Miller, Todd K. Hartman, Liat Levita, Liam Mason, Anton P. Martinez, Ryan McKay, Thomas V. A. Stocks, Kate Bennett, Philip Hyland, Richard P. Bentall

**Affiliations:** 1 School of Psychology, Ulster University, Derry, Northern Ireland; 2 Department of Psychology, University of Sheffield, Sheffield, UK; 3 Sheffield Methods Institute, Department of Psychology, University of Sheffield, Sheffield, UK; 4 Division of Psychology and Language Sciences, University College London, London, UK; 5 Department of Psychology, Royal Holloway, University of London, London, UK; 6 Institute of Population Health, University of Liverpool, Liverpool, UK; 7 Department of Psychology, Maynooth University, Maynooth, Republic of Ireland

## Abstract

**Background:**

The current study argues that population prevalence estimates for mental health disorders, or changes in mean scores over time, may not adequately reflect the heterogeneity in mental health response to the COVID-19 pandemic within the population.

**Methods:**

The COVID-19 Psychological Research Consortium (C19PRC) Study is a longitudinal, nationally representative, online survey of UK adults. The current study analysed data from its first three waves of data collection: Wave 1 (March 2020, *N* = 2025), Wave 2 (April 2020, *N* = 1406) and Wave 3 (July 2020, *N* = 1166). Anxiety-depression was measured using the Patient Health Questionnaire Anxiety and Depression Scale (a composite measure of the PHQ-9 and GAD-7) and COVID-19-related posttraumatic stress disorder (PTSD) with the International Trauma Questionnaire. Changes in mental health outcomes were modelled across the three waves. Latent class growth analysis was used to identify subgroups of individuals with different trajectories of change in anxiety-depression and COVID-19 PTSD. Latent class membership was regressed on baseline characteristics.

**Results:**

Overall prevalence of anxiety-depression remained stable, while COVID-19 PTSD reduced between Waves 2 and 3. Heterogeneity in mental health response was found, and hypothesised classes reflecting (i) stability, (ii) improvement and (iii) deterioration in mental health were identified. Psychological factors were most likely to differentiate the improving, deteriorating and high-stable classes from the low-stable mental health trajectories.

**Conclusions:**

A low-stable profile characterised by little-to-no psychological distress (‘resilient’ class) was the most common trajectory for both anxiety-depression and COVID-19 PTSD. Monitoring these trajectories is necessary moving forward, in particular for the ~30% of individuals with increasing anxiety-depression levels.

## Introduction

In response to fears of a ‘tsunami’ of mental ill health caused by the COVID-19 pandemic (e.g. Roxby, 2020), numerous attempts have been made to estimate the impact of the pandemic on populations (Holmes et al., 2020), either through cross-sectional surveys or, in fewer cases, longitudinal studies. However, these studies have reported population summary levels of distress (prevalence rates or mean scores) and have therefore implicitly made the unlikely assumption that response to the pandemic is homogenous. Here we show that this assumption is false, a finding with important implications for both future research and for public health measures in this and future global emergencies.

With two exceptions (see below), all longitudinal studies of population mental health in the pandemic have reported summary data (prevalence or mean scores for the populations as a whole). Two studies have compared pre-peripandemic levels of psychological distress using data from an existing nationally representative, probability-based cohort study re-fielded for the purposes of collecting COVID-19 data. It was reported that ‘clinically significant levels of mental distress’ among UK adults increased from 19% (95% CI 18–20%) in 2018–19 to 27% (26–28%) in April 2020 (Pierce et al., 2020a). Similarly, a US study reported the population prevalence estimate of ‘psychological distress’ among adults surveyed in April 2020 at 14% (11–17%), a substantial increase from the 4% (3.6–4.2%) reported in the 2018 National Health Interview Survey (McGinty, Presskreischer, Han, & Barry, 2020b). While these studies benefited from their use of probability-based samples, they used measures of psychological distress that are not diagnostic-specific (GHQ-12 and Kessler-6, respectively) and, in both cases, unavoidable changes in the mode of survey administration (e.g. from face-to-face to web/telephone-based assessments) between pre- and peri-pandemic assessments (Burton, Lynn, & Benzeval, 2020; McGinty et al., 2020b; Pierce et al., 2020a) limit the ability to draw comparisons from earlier waves. Furthermore, different parts of the USA became impacted at different times, there were differences across states in the severity of the lockdowns, and the locations of lockdowns were associated with local prevalence rates, and these factors make the interpretation of a single prevalence estimate very difficult.

Other studies have reported longitudinal comparisons between early stages in the pandemic and later time points. For example, McGinty, Presskreischer, Anderson, Han, and Barry (2020a) reported no statistically significant difference in the proportion of US respondents reporting serious psychological distress in April (14%; 95% CI 11–18%) *v*. July 2020 (13%; 10–17%). Hyland et al. (2021) reported similar results; the prevalence of generalised anxiety and depression did not significantly change between March (20%; 18–22% and 23%; 20–25%, respectively) and May 2020 (17%; 15–20% and 24%; 21–28%, respectively), during a nationwide lockdown in Ireland. During the same time period, a decrease in the population prevalence of generalised anxiety was reported in the UK, while depression remained stable (O'Connor et al., 2020). Using a much larger convenience sample weighted to match the population, Fancourt, Steptoe and Bu at University College London reported a decline in generalised anxiety and depression over the first 20 weeks of lockdown, with the greatest decline in the first 2 weeks (Fancourt, Steptoe, & Bu, 2021). As reported by other researchers (e.g. Shevlin et al., 2020), being younger, female, with children at home, having pre-existing mental health conditions and low income predicted high levels of depression and anxiety at the start of lockdown.

While the observation of heightened prevalence of common psychiatric disorders in the early stages of lockdown, which ameliorated with the passage of time, is an important counter-narrative to media reports of a ‘tsunami’ of mental ill-health, it seems unrealistic to assume that a single profile of longitudinal change will be found for the entire population; more likely, there will be different patterns of change, or ‘different slopes for different folks’. Indeed, it is known that many factors influence the likelihood of experiencing a psychiatric disorder. In the UK, for example, the *Adult Psychiatric Morbidity Survey* has reported that the prevalence of common mental disorders differs significantly according to socio-demographic factors such as age, gender, household type, employment, region of residence, and previous mental and physical health problems, so it seems plausible that some of these variables will affect the way that people react to the pandemic (McManus, Bebbington, Jenkins, & Brugha, 2016). In this context, it is important to note that overall prevalence levels or other summary scores are of little public health utility, as they cannot indicate where mental health service resources should be directed.

To our knowledge, only two short-term studies have tested heterogeneity of mental health response during the crisis. In a 6-week study of their large convenience sample, the UCL group (Iob, Frank, Steptoe, & Fancourt, 2020) reported three short-term trajectories for depressive (PHQ-9) symptoms using latent growth mixture modelling: a class with low depression (60.0%), a class with consistently moderate symptoms (29.0%) and a class with severe symptoms that decreased immediately after lockdown but then increased towards the end of the follow-up period (11.0%). In a sample of 523 German citizens already participating in a longitudinal study of resilience, Ahrens et al. (2021) found that both daily hassles and mean scores on the GHQ-28 decreased over the first 8 weeks of lockdown. Using latent growth mixture models, the authors then partitioned their sample into three groups: those with poor mental health which worsened over the first 3 weeks and then ameliorated afterwards (8.3%), a group that showed deterioration in mental health from the third week onwards (8.1%) and a majority (83.6%) whose mental health improved over the lockdown period. The short time period covering only the start of the pandemic in the UK, and the use of non-diagnostic measurements are serious limitations that our study aimed to rectify.

This study analyses panel data from three waves of a nationally representative sample of UK adults collected between March and July 2020. We tested three research questions related to the course of mental health difficulties during the introduction and subsequent easing of first lockdown restrictions within the UK.

The first research question was to determine if clinically relevant levels of anxiety-depression and COVID-19 posttraumatic stress disorder (PTSD) significantly changed over the first 4 months of the pandemic. We predicted that, overall, prevalence (and severity) will have declined in the Wave 3 (W3) survey due to (i) the easing of lockdown measures, (ii) the subsequent decline in the severity of the pandemic and (iii) adaption to living with pandemic-related restrictions, e.g. social distancing. Second, to test if there was significant heterogeneity at Wave 1 (W1), and if there were different longitudinal profiles of psychological distress over time. It was predicted that the 4-month mental health status of the UK population, assessed over three time points, will be represented by trajectories reflecting: (i) mental health *improvements* since the beginning of lockdown (recovery class), (ii) *deterioration* in mental health since the beginning of lockdown (deterioration class) and (iii) *stability* (no improvement or deterioration) since the beginning of lockdown.

Third, we aimed to identify which demographic, social, economic and psychological factors were associated with the different longitudinal profiles. These variables were chosen as they had been previously found to be related to pandemic-related psychopathology (Hyland et al., 2021; Shevlin et al., 2020) or had been considered and pre-registered as theoretically important (McBride et al., 2020a, 2020b). We predicted that trajectories reflecting poor or worsening mental health status will be associated with demographic variables (female gender, younger age, non-white ethnicity, lower income, living in a single adult household, living with dependent children, pre-existing mental health difficulties, living in an urban area), COVID-19-specific variables (lost income as a result of the pandemic, individual and family member chronic health condition, high perceived risk of being infected in the next month, individual or family member having been infected, individual or family member pregnancy) and psychological variables (higher levels of loneliness, death anxiety, intolerance of uncertainty , lower levels of resilience and an external locus of control). The study protocol and hypotheses were pre-registered before any W3 data analysis was conducted (https://osf.io/zheqt).

## Methods

### Sample

The COVID-19 Psychological Research Consortium (C19PRC) Study is a longitudinal, internet-based survey, designed to assess the UK population's psychological and social adjustments to the pandemic. Quota sampling methods ensured that the sample was representative of the UK adult population in terms of age, gender and gross household income. W1 (23–28 March 2020, *N* = 2025) recruited participants during the first week of the first UK lockdown. These individuals were followed-up approximately one month later (22 April–1 May 2020, *N* = 1406) for the Wave 2 (W2) survey, and again between 9 and 23 July 2020 (*N* = 1166) for W3. A detailed methodological account of the C19PRC Study is available elsewhere (McBride et al., 2020a, 2020b). Ethical approval was granted by the University of Sheffield (Ref. 033759). All participants provided informed consent.

### Measures

*Anxiety-depression*: The Patient Health Questionnaire Anxiety-Depression Scale (PHQ-ADS) is a 16-item scale comprising the PHQ-9 and GAD-7 used as a composite measure of depression and anxiety (Kroenke et al., 2016). Respondents were asked how often, over the past 2 weeks, they had been bothered by each of the depressive (nine items) and anxiety (seven items) symptoms. Responses are scored on a four-point Likert scale (0 ‘not at all’ to 3 ‘nearly every day’). Scores range from 0 to 48, with higher scores indicating higher levels of anxiety-depression symptomology. Moderate severity (20–48) was used to identify caseness, and scores from the PHQ-ADS have been found to demonstrate high internal reliability, as well as good convergent and construct validity in clinical samples (Kroenke, Baye, & Lourens, 2019; Kroenke et al., 2016).

*COVID-19-related PTSD*: The International Trauma Questionnaire is a self-report measure of ICD-11 PTSD based on a total of six symptoms across the three symptom clusters of Re-experiencing, Avoidance and Sense of Threat (Cloitre et al., 2018). Participants were asked to complete the ITQ ‘in relation to [their] experience of the COVID-19 pandemic…[and] how much [they] have been bothered by that problem in the past month’. The PTSD symptoms are accompanied by three items measuring functional impairment caused by these symptoms. All items are answered on a five-point Likert scale, ranging from 0 (Not at all) to 4 (Extremely) with possible PTSD scores ranging from 0 to 24. A score of ⩾2 (Moderately) is considered ‘endorsement’ of that symptom. A probable PTSD diagnosis requires at least one symptom to be endorsed from each PTSD symptom cluster, and endorsement of at least one indicator of functional impairment. The psychometric properties of the ITQ scores have been demonstrated in multiple general population (Ben-Ezra et al., 2018) and clinical and high-risk samples (Hyland et al., 2017).

A series of predictor variables were extracted from W1 as follows: age, gender, ethnicity, household income, urbanicity, employment, number of adults in the household, children present in the home and history of mental health treatment. Respondents were also asked whether they had lost income due to the pandemic, if they or a close family member had a chronic illness, their perceived risk of COVID-19 infection, COVID-19 infection status (self and other), and if they or a family member were pregnant. Psychological variables were also extracted: loneliness, resilience, locus of control, death anxiety and IU. See online Supplementary Material Section 1.1 for full details of predictor variables.

### Data analysis

Data analysis was undertaken in three linked phases. First, mean scores on the PHQ-ADS and ITQ were estimated for each survey time point, and tests for mean differences were conducted. Similarly, the proportion of participants scoring above the clinical cut-off score on the PHQ-ADS (⩾20), and those identified as cases by applying the ITQ diagnostic algorithm, were calculated for each wave, and the differences were tested for statistical significance. Further details outlining the steps involved in this process are included in online Supplementary Material Section 1.2.

The second phase of analysis used latent variable mixture modelling to identify different trajectories of change separately for anxiety-depression and COVID-19 PTSD (Muthén & Muthén, 2000; Muthén & Shedden, 1999). The baseline model in both cases was a latent growth model (LGM) with three observed variables representing the repeated measurements of both anxiety-depression and COVID-19 PTSD. This model was tested with the residual error variances constrained to be equal. The loadings on the intercept latent variable were fixed at 1, so the mean of the latent variable represented the average anxiety-depression and COVID-19 PTSD scores at W1. If the variance of the intercept latent variable is significant, then the hypothesis that all participants had the same level of anxiety-depression and COVID-19 PTSD at W1 can be rejected. The loading for the slope of the latent variable was fixed at 0, 1 and 2 to represent linear change over time. The mean of this latent variable represents the rate of change in anxiety-depression and COVID-19 PTSD over time. If the variance of the slope of the latent variable is statistically significant, this indicates that there is variability in participants' rate of change in psychological distress over time.

Significant variability in the intercept and slope indicates heterogeneity in the initial status and rate of change of anxiety-depression and COVID-19 PTSD levels among the participants. In this case, the heterogeneity can be modelled by adding a mixture component to the model to test if there are homogenous sub-groups of adults who share similar levels of initial status and rate of change of psychological distress. To accomplish this, latent class growth analysis (LCGA) was used to model the longitudinal trajectories (Nagin, 1999). LCGA is a restrictive form of a growth mixture model that specifies zero within-class variation for the intercept and slope latent variables and a slope-intercept correlation of zero; however, the means of the slope and intercept latent variables are allowed to vary across classes. LCGA involves adding latent classes successively to the LGM, with 1–7 class models being estimated. The parameters of the LGM and LCGA were estimated using full information robust maximum likelihood estimator to account for any missing data (Schafer & Graham, 2002). For additional information, refer to online Supplementary Material Section 1.3.

The third phase of analysis was to add predictors, or auxiliary variables, to the LCGA model, to assess which variables predict class membership. A three-step approach was taken where the inclusion of the predictors did not influence the formation of the classes (Kim, Vermunt, Bakk, Jaki, & Van Horn, 2016). This approach incorporates the classification uncertainties in the mixture model and has been shown to produce more accurate parameter estimates than other approaches that do not account for error in classification (Asparouhov & Muthén, 2014).

## Results

Table 1 presents the estimated mean PHQ-ADS and ITQ scores, and proportions of the sample meeting the clinical criteria, at each wave. The PHQ-ADS mean scores were similar from W1 to W3, and the equality test indicated that there were no significant differences, so the level of anxiety-depression remained stable across this time period. The percentage of participants meeting the clinical criteria for anxiety-depression was 20.7% at W1, and there was no significant change at W2 (18.6%) or W3 (20.0%). The ITQ mean scores were similar at W1 (M = 4.58) and W2 (M = 4.51), but decreased at W3 (M = 4.07); pairwise comparisons showed that the mean at W3 was significantly lower than the mean at W1 [Wald χ^2^ (1) = 12.02, *p* < 0.001] and W2 [Wald χ^2^ (1) = 8.78, *p* < 0.001]. The percentage of the sample that met the criteria for COVID-19 PTSD also decreased across time, the only significant pairwise comparison was between W1 and W3 [Wald χ^2^ (1) = 5.64, *p* < 0.001].

**Table 1.** Means scores and caseness for anxiety-depression (PHQ-ADS) and traumatic stress (ITQ)

	Wave 1	Wave 2	Wave 3	
	Mean (s.e.)	Mean (s.e.)	Mean (s.e.)	Equality test
Anxiety-depression: PHQ-ADS	10.53 (0.25)	10.33 (0.28)	10.68 (0.31)	χ^2^(2) = 2.40, *p* = 0.301
Traumatic stress: ITQ	4.58 (0.13)	4.51 (0.15)	4.07 (0.15)	χ^2^(2) = 15.88, *p* < 0.001
	% (95% CI)	% (95% CI)	% (95% CI)	
Anxiety-depression: PHQ-ADS	20.7% (19.0–22.2)	18.6% (16.6–20.6)	20.0% (17.8–22.2)	χ^2^(2) = 5.20, *p* = 0.074
Traumatic stress: ITQ	16.8% (15.2–18.4)	15.8% (13.9–17.6)	14.4% (12.4–16.3)	χ^2^(2) = 6.23, *p* = 0.042

PHQ-ADS, The Patient Health Questionnaire Anxiety-Depression Scale; ITQ, International Trauma Questionnaire; s.e., standard error.

The baseline LGMs with equal residual error variances for anxiety-depression and PTSD indicated that the variance of the latent variables for the intercepts and slopes was significant. This meant that heterogeneity could be explored using LCGA. Full details of the LGM results are included in online Supplementary Material (Section 1.4). Online Supplementary Tables S1 and S2 show the fit indices for the LCA models of anxiety-depression and traumatic stress, respectively. In both models, the information theory-based fit statistics decreased from 1 to 7 class models, the largest difference in the BIC was evident between the 4 and 5 class solution (anxiety-depression ΔBIC = 215.89; COVID-19 PSTD ΔBIC = 215.98). The entropy of the 5-class solution indicated a high level of correct classification for both anxiety-depression (0.81) and COVID-19 PSTD (0.86). The LMR-A was non-significant for the 6-class solution. Collectively, these findings support the selection of the 5-class model as the optimal solution.

**Fig. 1.**
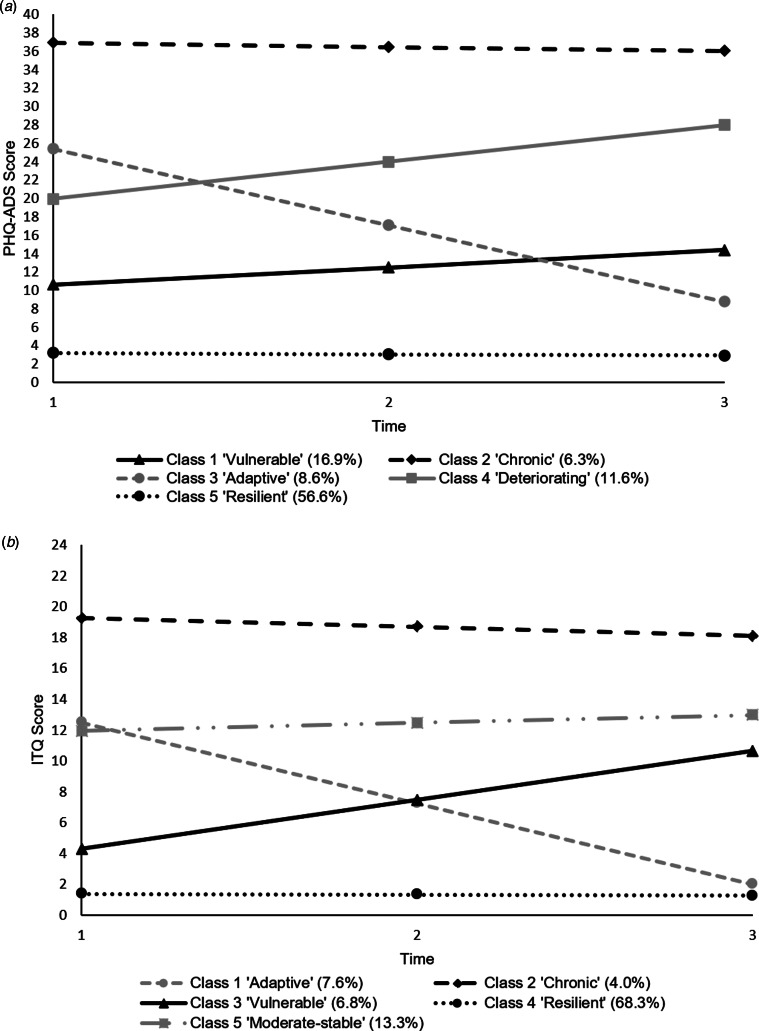
Profile plots of the longitudinal trajectories of (*a*) the 5-class model of anxiety-depression and (*b*) the 5-class model of COVID-19 PTSD.

Table 2 displays the parameter estimates for the 5-class LCGA models for anxiety-depression and traumatic stress, and the trajectories are shown in Fig. 1. For anxiety-depression, Class 5 was the largest (56.6% of the sample) and was defined by low baseline anxiety-depression mean and a very shallow decrease (the slope is significant but represents a decrease of less than a fifth of a scale point between intervals); this is the ‘resilient class’. Class 2 (6.3%) had a high baseline mean score and was stable over time (the slope was not significant); this is the ‘chronic class’. Classes 3 (8.6%) and 4 (11.6%) had similar moderate baseline scores, but the rate of change was greater for Class 3 (‘adaptive class’) compared to Class 4 (‘deteriorating class’). Class 1 (16.9%) had a low-moderate baseline mean score, and a significant increase over time (‘vulnerable class’). Similar trajectories were found for COVID-19 PTSD; a ‘chronic class’ (4.0%), a ‘resilient class’ (68.3%), an ‘adaptive class’ (7.6%), a ‘vulnerable class’ (6.8%). While a deteriorating anxiety-depression class emerged, a corresponding class for COVID-19 PTSD did not increase over time, instead it had a similar level at W1, i.e. a ‘moderate-stable class’ (13.3%).

**Table 2.** Class-specific parameter estimates for the 5-class models of anxiety-depression and traumatic stress

	Anxiety-depression				
	Vulnerable	Chronic	Adaptive	Deteriorating	Resilient
Intercept mean (s.e.)	10.618 (0.643)***	36.959 (0.951)***	25.399 (1.122)***	19.969 (1.770)***	3.211 (0.191)***
Slope mean (s.e.)	1.877 (0.715)**	−0.508 (0.618)	−8.315 (0.867)***	4.010 (1.062)***	−0.154 (0.076)*
*N* (%)	343 (16.9%)	127 (6.3%)	174 (8.6%)	235 (11.6%)	1146 (56.6%)
	Traumatic stress				
	Vulnerable	Chronic	Adaptive	Moderate-stable	Resilient
Intercept mean (s.e.)	4.285 (0.487)***	19.275 (0.657)***	12.505 (0.597)***	11.960 (0.442)***	1.388 (0.072)***
Slope mean (s.e.)	3.180 (0.729)***	−0.571 (0.649)	−5.250 (0.385)***	0.508 (0.446)	−0.054 (0.041)
*N* (%)	137 (6.8%)	81 (4.0%)	154 (7.6%)	270 (13.3%)	1383 (68.3%)

s.e., standard error.

Table 3 shows that, compared to Class 5 (‘resilient class’), having had mental health treatment, and higher levels of loneliness, death anxiety and IU all increased the likelihood of membership of all other anxiety-depression classes. The odds ratios were highest for the ‘chronic’ class. Higher levels of resilience were associated with decreased likelihood of membership of all anxiety-depression classes compared to Class 5 (‘resilient’). There were also some class-specific associations. The ‘vulnerable’ class was associated with someone close having a chronic illness and a high perceived risk of being infected with COVID-19. The ‘chronic’ class was associated with being male, lower income, having a chronic illness and low internal locus of control. The ‘adaptive’ class was associated with having lost income, having a chronic illness and a high perceived risk of being infected with COVID-19.

**Table 3.** Predictors (odds ratios) of anxiety-depression trajectories

	Vulnerable	Chronic	Adaptive	Deteriorating
Gender (female)	1.06 (0.67–1.68)	0.29* (0.13–0.65)	1.54 (0.82–2.9)	0.89 (0.52–1.53)
Age	0.99 (0.97–1.01)	0.97 (0.94–1.00)	0.99 (0.96–1.01)	0.99 (0.97–1.01)
Ethnicity (white)	1.06 (0.43–2.64)	1.78 (0.55–5.76)	1.46 (0.49–4.31)	0.73 (0.33–1.62)
Economically active	0.84 (0.5–1.41)	0.80 (0.36–1.75)	1.03 (0.48–2.20)	0.98 (0.53–1.81)
Income category	1.06 (0.89–1.27)	0.71* (0.52–0.95)	1.15 (0.9–1.45)	0.94 (0.77–1.16)
Lost income	1.41 (0.89–2.25)	1.90 (0.93–3.9)	2.35* (1.32–4.18)	1.19 (0.69–2.04)
Lone adult	0.67 (0.37–1.2)	0.36* (0.13–0.97)	0.99 (0.43–2.28)	0.86 (0.42–1.75)
Children at home	0.71 (0.42–1.19)	0.95 (0.44–2.04)	1.17 (0.57–2.39)	1.27 (0.69–2.34)
Urban	0.75 (0.44–1.27)	1.16 (0.56–2.39)	0.61 (0.28–1.31)	1.71 (0.98–2.99)
Mental health treatment	3.65* (2.24–5.93)	12.83* (5.16–31.93)	2.92* (1.5–5.68)	5.44* (3.18–9.28)
Chronic illness – self	0.78 (0.37–1.64)	2.87* (1.03–7.97)	2.31* (1.09–4.87)	0.77 (0.33–1.79)
Chronic illness – close	1.92* (1.17–3.15)	1.17 (0.48–2.82)	1.83 (0.92–3.64)	1.24 (0.64–2.40)
COVID-19 infection – self	0.94 (0.18–4.81)	4.99 (0.87–28.65)	3.38 (0.91–12.58)	0.50 (0.05–5.31)
COVID-19 infection – other	1.77 (0.63–4.94)	2.56 (0.66–9.95)	2.43 (0.68–8.71)	2.6 (0.81–8.37)
Pregnant	1.70 (0.43–6.68)	1.09 (0.19–6.27)	0.69 (0.15–3.20)	1.47 (0.33–6.66)
Family pregnant	0.56 (0.16–1.99)	0.95 (0.22–4.16)	1.01 (0.27–3.70)	0.65 (0.21–1.95)
Loneliness total	1.66* (1.42–1.93)	2.95* (2.25–3.87)	1.69* (1.41–2.02)	1.94* (1.64–2.29)
Resilience total	0.92* (0.86–0.98)	0.82* (0.75–0.90)	0.86* (0.8–0.93)	0.90* (0.84–0.96)
Locus of control – internal	0.93 (0.87–1.00)	0.87* (0.78–0.97)	0.94 (0.84–1.04)	0.86* (0.80–0.93)
Locus of control – chance	0.97 (0.9–1.05)	1.02 (0.89–1.17)	1.01 (0.88–1.14)	0.98 (0.89–1.08)
Locus of control – powerful others	1.01 (0.94–1.09)	1.13* (1.01–1.27)	1.09 (0.98–1.21)	1.03 (0.94–1.12)
Death anxiety	1.02* (1.00–1.04)	1.04* (1.01–1.07)	1.03* (1.01–1.06)	1.03* (1.01–1.05)
Intolerance of uncertainty	1.05* (1.02–1.09)	1.13* (1.07–1.19)	1.09* (1.04–1.14)	1.08* (1.04–1.12)
Perceived COVID risk – medium	1.38 (0.82–2.33)	0.84 (0.34–2.06)	2.02 (0.81–5.01)	0.79 (0.43–1.46)
Perceived COVID-19 risk – high	2.15* (1.18–3.93)	2.62 (0.96–7.15)	3.77* (1.47–9.68)	1.41 (0.68–2.90)

*Note*: *p < .05. Reference class is ‘resilient’ anxiety-depression trajectory.

Table 4 shows that, compared to COVID-19 PSTD Class 4 (‘resilient’ class), all other COVID-19 PTSD class members were generally associated with higher levels of loneliness, external locus of control (powerful others) and death anxiety. The moderate baseline-stable class was associated with being male, having children at home, living in an urban area, mental health treatment history, someone close having COVID-19, lower internal locus of control and a high perceived risk of being infected with COVID-19. The ‘adaptive’ class was also associated with being economically active and higher IU.

**Table 4.** Predictors (odds ratios) of traumatic stress trajectories

	Adaptive	Chronic	Vulnerable	Moderate-stable
Gender (female)	0.71 (0.39–1.29)	0.36 (0.10–1.32)	0.85 (0.51–1.4)	0.56* (0.33–0.96)
Age	0.99 (0.96–1.01)	0.98 (0.92–1.03)	1.01 (0.99–1.03)	0.96* (0.93–0.98)
Ethnicity (white)	0.56 (0.25–1.26)	1.56 (0.43–5.72)	0.57 (0.21–1.56)	0.56 (0.29–1.11)
Economically active	2.10* (1.10–4.04)	5.06 (0.74–34.8)	1.45 (0.82–2.58)	1.66 (0.93–2.94)
Income category	1.13 (0.94–1.37)	1.08 (0.62–1.89)	1.04 (0.87–1.25)	0.93 (0.75–1.14)
Lost income	0.81 (0.46–1.44)	2.10 (0.61–7.27)	1.04 (0.61–1.78)	1.34 (0.81–2.22)
Lone adult	1.12 (0.55–2.27)	1.53 (0.42–5.63)	0.95 (0.46–1.97)	1.55 (0.82–2.93)
Children at home	1.53 (0.85–2.73)	2.24 (0.82–6.08)	0.97 (0.49–1.92)	1.74* (1.01–3.01)
Urban	1.06 (0.54–2.06)	2.05 (0.45–9.35)	1.11 (0.61–2.02)	1.74* (1.05–2.86)
Mental health treatment	1.23 (0.65–2.30)	5.00 (0.93–26.81)	1.71 (0.94–3.09)	1.99* (1.16–3.41)
Chronic illness – self	1.47 (0.6–3.62)	4.06 (0.47–35.3)	0.93 (0.44–1.96)	1.18 (0.55–2.54)
Chronic illness – close	0.47* (0.22–0.99)	0.66 (0.22–1.98)	1.16 (0.64–2.10)	1.52 (0.85–2.72)
COVID-19 infection – self	1.42 (0.19–10.43)	2.45 (0.14–43.52)	1.06 (0.22–5.10)	3.96* (1.58–9.90)
COVID-19 infection – other	0.84 (0.22–3.2)	0.79 (0.12–4.96)	1.98 (0.65–5.99)	2.78* (1.16–6.66)
Pregnant	1.95 (0.49–7.79)	0.40 (0.05–3.20)	0.45 (0.03–6.60)	1.16 (0.28–4.83)
Family pregnant	1.28 (0.41–4.03)	0.64 (0.02–16.95)	0.47 (0.06–3.63)	0.86 (0.24–3.09)
Loneliness total	1.34* (1.15–1.56)	1.71* (1.24–2.36)	1.30* (1.08–1.56)	1.43* (1.25–1.65)
Resilience total	0.95 (0.89–1.00)	0.93 (0.78–1.12)	0.97 (0.89–1.04)	0.98 (0.92–1.04)
Locus of control – internal	0.91 (0.81–1.02)	1.12 (0.74–1.68)	0.92 (0.84–1.00)	0.85* (0.77–0.93)
Locus of control – chance	1.06 (0.96–1.19)	1.11 (0.86–1.43)	1.00 (0.91–1.11)	0.92 (0.84–1.02)
Locus of control – powerful others	1.18* (1.06–1.3)	1.41* (1.17–1.69)	1.04 (0.95–1.14)	1.20* (1.09–1.32)
Death anxiety	1.05* (1.03–1.08)	1.10* (1.03–1.18)	1.03* (1.00–1.05)	1.06* (1.04–1.08)
Intolerance of uncertainty	1.05* (1.00–1.10)	1.05 (0.99–1.13)	1.05* (1.01–1.1)	0.99 (0.95–1.03)
Perceived COVID risk – medium	0.60 (0.31–1.15)	3.00 (0.79–11.35)	1.22 (0.67–2.21)	1.97 (0.93–4.21)
Perceived COVID-19 risk – high	1.30 (0.69–2.45)	4.87 (0.91–26.1)	1.86 (0.93–3.74)	3.90* (1.75–8.69)

*Note*: *p < .05. Reference class is ‘resilient’ traumatic stress trajectory.

## Discussion

The current study attempted to overcome an important limitation present within the majority of COVID-19 mental health literature to date: failure to account for heterogeneity in psychological response to the outbreak, which may undermine the ability to accurately identify groups of individuals most in need of support. The current findings suggest that for the overall sample, the prevalence of anxiety-depression remained stable across the first 4 months of the pandemic, while COVID-19-related PTSD fell between April and July 2020. Despite being elevated and stable, the prevalence of anxiety-depression reported does not appear to be markedly higher than in previous epidemiological surveys (Shevlin et al., 2020). The overall decrease in COVID-19-related PTSD between W2 and W3 may be suggestive of habituation to the situation, causing individuals to be less ‘alert’ to the virus, or reduced the frequency of upsetting COVID-19 imagery in the media.

Like the findings of the UCL group (Iob et al., 2020) and of Ahrens et al. (2021) over shorter periods, our findings refute the null hypothesis that the population response to the pandemic was homogeneous. For both anxiety-depression and COVID-19 PTSD, hypothesised classes representing (i) stability, (ii) improvement and (iii) deterioration in mental health severity emerged. As predicted, the majority of the sample exhibited resilient mental health trajectories (anxiety-depression, 56.6%; COVID-19 PTSD, 68.3%) characterised by minimal changes in anxious-depressive or PTSD symptomology during the earliest months of the pandemic (March–July 2020). This aligns with previous research which suggests that, although some individuals may exhibit long-term distress following traumatic/adverse events, resilience (maintaining healthy outcomes or ‘bouncing back’ following such events) is the most common and consistently observed response (Bonanno, 2004; Galatzer-Levy, Huang, & Bonanno, 2018; Goldmann & Galea, 2014).

For both mental health outcomes, around 8% of individuals belonged to classes displaying improvement over the 4-month period (anxiety-depression, 8.6%; COVID-19 PTSD, 7.6%). Based on the cut-off points of PHQ-ADS severity, the adaptive class trajectory moved from the ‘moderate’ to ‘mild’ range. However, a small group of individuals exhibited severe psychological distress throughout the first months of lockdown (anxiety-depression, 6.3%; COVID-19 PTSD, 4.0%), and classes also emerged displaying trajectories of deterioration. Concerningly, for anxiety-depression, this included a deteriorating group (11.6%) and a vulnerable group (16.9%); for COVID-19 PTSD, there was a corresponding vulnerable group only (6.8%). A moderate-stable COVID-19 PTSD class also emerged (13.3%) however. The emergence of both improving and deteriorating classes in the current study suggests that while it may have taken several months for some individuals to adjust and adapt to the situation, for others, deterioration may have only emerged after months of increased caring duties, balancing home and work life, or with the end of the furlough scheme looming.

Broadly, our findings suggest that individuals with a history of mental health treatment, higher levels of loneliness, IU, death anxiety and external locus of control, and lower levels of resilience were more likely to be a member of the anxiety-depression/COVID-19 PTSD trajectories characterised by some degree of psychological distress, compared to those in the ‘resilient’ trajectories. The finding that these predictors were associated with improving, deteriorating and stable trajectories suggests it is likely that these variables, measured at the earliest stage of the pandemic, were predicting individuals' trajectory intercepts rather than their slopes (i.e. all starting with some degree of psychological distress). As such, further analysis is needed to examine how a change in these variables over time affects change in mental health status. Many of the most consistently reported demographic and COVID-19-specific predictors of distress during this period (e.g. female gender, younger age, living with children, having a physical or mental health condition) were less consistently associated across classes in the current study (Hyland et al., 2021; Iob et al., 2020; O'Connor et al., 2020; Pierce et al., 2020a), although there were some unique class-specific predictors.

In addition to accounting for heterogeneity in psychological response, additional strengths of this study include its nationally representative sample, use of preferred ‘gold standard’ diagnostic-specific measures of depression and anxiety, pre-registered hypotheses and use of data across three time points which capture the pre-peak, peak and post-peak stages of the first coronavirus wave in the UK. Furthermore, consistent mode of survey administration and assessment allows for accurate between-waves comparisons. In particular, the results are not compromised by social desirability bias, with these effects being lower for online completed surveys compared to face-to-face administration (Zhang, Kuchinke, Woud, Velten, & Margraf, 2017). Several study limitations, however, should be acknowledged. First, the current study was not a true random probability sample, which, given the circumstances and restrictions since the study inception, would be difficult to achieve. Non-probability surveys have been criticised as being biased towards both over- and under-inclusion of psychologically distressed individuals (Chauvenet, Buckley, Hague, Fleming, & Brough, 2020; Pierce et al., 2020b) and it is conceivable that psychological factors influenced the decision to participate in the survey, creating a possibility of sampling bias. Second, with data from only three time points, some restrictions had to be placed on the model, specifically the within-class slope and intercept variability was constrained to zero. Some evidence has shown that this approach may lead to over-extraction of classes (Bauer & Curran, 2003; Diallo, Morin, & Lu, 2016) and overestimate the size of the baseline, or ‘resilient’ class (Infurna & Luthar, 2018), and so the solutions should be interpreted with this in mind.

Continued investigation as to how these trajectories develop is necessary moving forward, particularly in light of the reinstatement of more stringent restrictions and a second peak in COVID-19 cases during autumn/winter 2020. In particular, it will be important to monitor those currently within trajectories of increasing distress (anxiety-depression: ~30%; COVID-19 PTSD: ~7%). A more detailed understanding of the factors that influence these trajectories is also needed, specifically, accounting for change in many factors as a result of the current situation (e.g. infection status, employment, etc.).

Investigation of these trajectories is likely to have considerable implication for public health efforts; although summary scores may be a useful starting point for this purpose, they are potentially misleading because they fail to distinguish between those who have chronic and pre-existing mental health difficulties (likely the majority in the chronic classes in our analyses), those who are coping well or benefiting from the changing circumstances (the resilient and adaptive classes) and new cases of distress that have been provoked by the pandemic (the vulnerable and deteriorating classes). In a time in which national economic resources are challenged, and in which health care services may be under considerable pressure, it is clearly important that public health interventions should be directed to those who most likely to be harmed by the pandemic, and not to those who are unaffected. Furthermore, the predictors of class membership may provide clues about the type of mass interventions that are likely to be effective, which may extend beyond conventional therapeutic services. For example, it is notable that loneliness appears to be an important factor, which is consistent with the evidence that social engagement confers resilience to common psychiatric disorders (Haslam, Jetten, Postmes, & Haslam, 2009); hence interventions that promote engagement between neighbours are likely to be especially helpful during times of lockdown. Similarly, the observation that poor locus of control and IU predict poor coping suggests that government advice and messaging should be directed towards addressing these vulnerabilities. Indeed, in times of severe threat to the health and wellbeing of the nation, it is vital that all aspects of government activity geared towards protecting citizens – for example, strategies for delivering testing, advice about work and social distancing and, of course, economic relief – are evaluated for their mental health implications in advance.

## Supplementary material

For supplementary material accompanying this paper visit http://dx.doi.org/10.1017/S0033291721001665. click here to view supplementary material
