# Supplementary material for: Refuting the myth of a ‘tsunami’ of mental ill-health in populations affected by COVID-19: evidence that response to the pandemic is heterogeneous, not homogeneous
Source: Psychol Med. 2021 Apr 20:1–9. doi: 10.1017/S0033291721001665 (PMC8111207; doi:10.1017/S0033291721001665)
Supplement: Supplementary file 1 [file S0033291721001665sup001.docx]

**Online supplementary material for ‘Refuting the myth of a ‘tsunami’ of mental ill-health in populations affected by COVID-19: Evidence that response to the pandemic is heterogenous, not homogeneous’ Shevlin et al. (2021)**

This online supplement contains further details of predictor variables used in the regression analyses, statistical methods, and procedures for assessing goodness of fit.

- 1. **Details of predictor variables**

A series of predictor variables were extracted from W1 as follows:

Demographic variables of age (years), gender (female=1; male = 0).

Ethnicity was recoded into a binary variable (1= White British/Irish or White non-British/Irish; 0 = Indian, Pakistani, Chinese, Afro-Caribbean, African, Arab, Bangladeshi, Other Asian, or Other ethnic group).

Income: Participants were asked “Please choose from the following options to indicate your approximate gross (before tax is taken away) household income in 2019 (last year). Include income from partners and other family members living with you and all kinds of earnings including salaries and benefits” to choose one of 5 categories: “£0 - £300 per week (equals about £0 - £1290 per month or £0 - 15,490 per year)”, “£301 - £490 per week (equals about £1,291 - £2,110 per month or £15,491 - £25,340 per year)”, “£491 - £740 per week (equals about £2,111 - £3,230 per month or £25,341 - £38,740 per year)”, “£741 - £1,111 per week (equals about £3,231 - £4,830 per month or £38,741 - £57,930 per year)”, and “£1,112 or more per week (equals about £4,831 or more per month or £57,931 or more per year)”.

Urbanicity: Participants were asked “Do you consider yourself to live in:” and were required to choose one of the options provided: ‘City’, ‘Suburb’, ‘Town’, or ‘Rural’. The variable was recoded to a binary variable representing urbanicity (1= City; 0 = Suburb, Town, or Rural).

Employment: Participants were asked to select if they were Employed full time, Employed part time, Unemployed looking for work, Unemployed not looking for work, Retired, Student or Disabled. These were recoded as binary variable with the first 2 options labelled ‘Economically active’ (1) and ‘Economically inactive’ (0).

Lone adult: Participants were asked “How many adults (18 years or above) live in your household (including yourself)?” and were provided with options ranging from ‘1’ to ’10 or more’. The data were recoded into a binary variable to represent lone adult household status (1 = lone adult; 0 = more than 1 adult in the household).

Children at home: Participants were asked “How many children (below the age of 18) live in your household?” and were provided with options ranging from ‘1’ to ’10 or more’. The scores were categorised into a binary variable (1= At least one dependent child in the household; 0 = No dependent children in the household).

Loss of income: Participants were asked “Some people have lost income because of the coronavirus COVID-19 pandemic, for example because they have not been able to work as much or because business contracts have been cancelled or delayed. Please indicate whether your household has been affected in this way” and the response options were “My household has lost income because of the coronavirus COVID-19 pandemic”, “My household has not lost income because of the coronavirus COVID-19 pandemic”, “I do not know whether my household has lost income because of the coronavirus COVID-19 pandemic”. The first option was considered as ‘Yes’ (1) and the other options were collapsed to represent ‘No’.

History of mental health treatment: Participants were asked “Mental health difficulties are very common. It will help us understand our survey results if you would tell us whether you currently or have in the past received treatment (medication or talking therapies) for these kind of difficulties”, and the response categories were ‘I have never received treatment for mental health problems’, ‘I have received treatment for mental health problems in the past’, ‘I'm currently receiving treatment for mental health problems’, and ‘I prefer not to answer this question’. The responses were recoded into a binary variable to represent ‘Mental health treatment (1= ‘I have received treatment for mental health problems in the past’ or ‘I'm currently receiving treatment for mental health problems’) and ‘No mental health treatment’ (0= ‘I have never received treatment for mental health problems’ or ‘I prefer not to answer this question’).

Chronic illness: Participants were asked “Do you have diabetes, lung disease, or heart disease?” and the response options were ‘Yes’ (1) and ‘No’ (0). They were also asked “Do any of your immediate family have diabetes, lung disease, or heart disease?” and the response options were ‘Yes’ (1) and ‘No’ (0).

Perceived risk of COVID-19 infection: Participants were asked “What do you think is your personal percentage risk of being infected with the COVID-19 virus in the next month?” Responses were collected on a slider scale which had ‘0’ and ‘100’ at the left and right hand extremes respectively, showed 10 point increments, and the labels ‘No Risk’, ‘Moderate Risk’ and ‘Great Risk’ were shown on the left, middle and right-hand part of the scale, respectively. This produced a continuous score ranging from 0 to 100 with higher scores reflecting higher levels of perceived risk of being infected by COVID-19. The scores were recoded into ‘Low’ (0 - 33), ‘Moderate’ (34 - 67), and ‘High’ (68 - 100).

COVID-19 status, self and other: Participants were asked “Have you been infected by the coronavirus COVID-19?” and six responses were provided. These were collapsed into a binary variable representing ‘COVID-19 infection’. Positive perceived infection status was based on the selection of either, ‘I have the symptoms of the COVID-19 virus and think I may have been infected’ or ‘I have been infected by the COVID-19 virus and this has been confirmed by a test’. Negative perceived infection status was based on the selection of either, ‘No. I have been tested for COVID-19 and the test was negative’, ‘No, I do not have any symptoms of COVID-19’, ‘I have a few symptoms of cold or flu but I do not think I am infected with the COVID-19 virus’ or ‘I may have previously been infected by COVID-19 but this was not confirmed by a test and I have since recovered’. Positive status (self) was coded ‘1’ and negative status coded as ‘0’. Participants were also asked “Has someone close to you (a family member or friend) been infected by the coronavirus COVID-19?” and four responses were provided. These were collapsed into a binary variable representing ‘Perceived infection status – someone close’. Positive perceived infection status was based on the selection of either, ‘Someone close to me has symptoms, and I suspect that person has been infected’ or ‘Someone who is close to me has had a COVID-19 virus infection confirmed by a doctor’. Negative perceived infection status was based on the selection of either, ‘No’ or ‘Someone close to me has symptoms, but I am not sure if that person is infected’. Positive status (other) was coded ‘1’ and negative status coded as ‘0’.

Pregnant: Participants were asked ‘Are you pregnant?’ and ‘Are any of your immediate family pregnant at this time?’ and responses were Yes (1) or No (0).

Loneliness: The three-item Loneliness Scale asks participants to indicate how often they feel they lack companionship, left out, and isolated from others (Hughes, Waite, Hawkley, & Cacioppo, 2004). Responses are scored using a three-point scale including (1) ‘Hardly Ever’, (2) ‘Sometimes’, and (3) ‘Often’. Possible scores range from 3 to 9 with higher scores indicating higher levels of loneliness. The internal reliability of the scale scores in this sample was good (*α* = .88).

Resilience: The Brief Resilience Scale (BRS) comprises six-items answered using a five-point Likert scale ranging from ‘Strongly Disagree’ (1) to ‘Strongly Agree’ (5) (Smith et al., 2008). Possible scores range from 6 to 30 with higher scores indicating higher levels of resilience. The internal reliability in this sample was good (*α* = .88).

Locus of control: The Locus of Control Scale (LoCS) measures three forms of locus of control: ‘Internal’, ‘Chance’, and ‘Powerful Others’ (Sapp & Harrod, 1993). Each subscale is based on three questions, and all questions use a seven-point Likert scale ranging from (1) ‘Strongly Disagree’ to (7) ‘Strongly Agree’. Higher scores reflect higher levels of each construct, and the internal reliability of the Internal’ (*α* = .71) and ‘Chance’ (*α* = .70) subscales were acceptable; the ‘Powerful Others’ (*α* = .85) subscale was good.

Death anxiety: The Death Anxiety Inventory (DAI) includes 17 items based on a five-point Likert scale ranging from (1) ‘Totally Disagree’ to (5) ‘Totally Agree’ (Tomás-Sábado, Gómez-Benito, & Limonero, 2005). Higher scores indicate higher levels of death anxiety. The internal reliability of the DAI scores in this sample was excellent (*α* = .94).

Intolerance of uncertainty: The Intolerance of Uncertainty scale (IUS) includes 12 items (answered using a five-point Likert scale ranging from (1) ‘Not at All Characteristic of Me’ to (5) ‘Entirely Characteristic of Me’(Buhr & Dugas, 2002). Higher scores indicate increased levels of intolerance of uncertainty. The internal reliability of the IUS scores in this sample was good (*α* = .86).

- 1. **Differences in PHQ-ADS and ITQ mean scores and clinical cut-off criteria across 3 waves**

This analytic process involves several steps. Initially, mean scores for each variable were fitted to a null or ‘constrained’ model; this included the three means, variances, and covariances, and the three means are constrained to be equal. Then, an ‘unconstrained’ model is specified without the equality constraint, allowing the three means to be freely estimated. The constrained and unconstrained models differ by two degrees of freedom, so improvement can be tested using the loglikelihood difference test, based on the chi-square (χ^2^) statistic. A significant χ^2^ value indicates that the unconstrained model is better than the constrained model, meaning that the null hypothesis of equal means can be rejected (Hoffman, 2015). Importantly, the use of robust maximum likelihood (MLR) estimation means that all available information at waves 1, 2 and 3 can be used to estimate the means, variances, and covariances, thus avoiding the deleterious effects of listwise deletion. This approach is analogous to a repeated measures analysis of variance (ANOVA); however, it does not make assumptions about the variance-covariance structure of the observations (Hoffman, 2015), and missing data are handled efficiently using full information MLR estimation (Schafer & Graham, 2002). These models were specified and estimated using Mplus Version 8.1 (Muthén & Muthén, 2018). Mplus also incorporates the ‘model test’ feature that allows specific constraints to be tested using the Wald χ^2^ test, and this was used to test pairwise comparisons: if the null hypothesis of equal means was rejected, then pairs of means were tested to determine which were significantly different. This approach was also used to test for the equality of proportions (i.e., changes probable diagnostic rates) across the three assessment periods for the PHQ-ADS and ITQ.

- 1. **Assessing the fit of latent class growth models**

To avoid solutions based on local maxima for the LCGA, 200 random sets of starting values were used initially followed by 50 final stage optimizations. The fit of the baseline LGM was assessed using standard criteria: acceptable fit was indicated by non-significant χ^2^, TLI and CFI greater than .90, RMSEA and SRMR <.08. The relative fit of the LCGA models was compared by using three information theory based fit statistics: The Akaike Information Criterion (AIC) (Akaike, 1987), the Bayesian Information Criterion (BIC) (Schwarz, 1978), and the sample size adjusted Bayesian Information Criterion (ssaBIC) (Sclove, 1987). The solution that produces the lowest value can be judged the best model, or if no minimum is found then the “diminishing gains in model fit” (p. 572) for additional classes can be examined (Masyn, 2013). This is analogous to the scree plot in exploratory factor analysis, where an ‘elbow’ in the values of the information criteria is evidenced, indicating that additional classes are making a minimal improvement in model fit for the cost of additional model complexity. Evidence from simulation studies have demonstrated that the BIC is the best information criterion for identifying the correct number of classes (Nylund, Asparouhov, & Muthén, 2007). In addition, the Lo-Mendell-Rubin adjusted likelihood ratio test (LMR-A) was used to compare models with increasing numbers of latent classes (Lo, Mendell, & Rubin, 2001). When a non-significant value (p > .05) occurs, this suggests that the model with one fewer class should be accepted.

- 1. **Additional details of the latent growth models**

The baseline LGMs with equal residual error variances for anxiety-depression (χ^2^ (3) = 4.90, p = .179; CFI = .99; TLI = .997; RMSEA = .018 (90% CI .000, .045); SRMR = .012) and traumatic stress (χ^2^ (3) = 5.24, p = .154; CFI = .996; TLI = .996; RMSEA = .019 (90% CI .000, .046); SRMR = .022) were both well-fitting models. For the anxiety-depression model, the latent variable intercept mean was 10.485 (s.e. = 0.248, p <.001), and the slope mean was 0.035 (s.e. = 0.125, p = .781); this suggests that the estimated overall mean at W1 was 10.485, and there was no change over time (as the slope mean was not significant). The variance of the latent variables for the intercept (σ^2^ = 98.54, s.e. = 4.93, p < .001) and slope (σ^2^ = 3.56, s.e. = 1.61, p < .05) were significant, and this indicted that there was heterogeneity that could be explored using LCGA. For the traumatic stress model, the latent variable intercept mean was 4.613 (s.e. = 0.128, p <.001), and the slope mean was -0.225 (s.e. = 0.071, p < .01); thus, the estimated overall mean at W1 was 4.613, and there was significant change over time with an estimated decrease of .225 points on the ITQ between each time interval. The variance of the latent variables for the intercept (σ^2^ = 24.771, s.e. = 1.332, p < .001) and slope (σ^2^ = 1.381, s.e. = 0.503, p < .01) were significant, and this indicted that there was heterogeneity that could be explored using LCGA.

**Table S1. Fit Indices for the Latent Class Growth Analysis for Anxiety-Depression.**

*AIC* Akaike Information Criterion; *BIC* Bayesian Information Criterion; *ssaBIC* sample size adjusted Bayesian Information Criterion; *LMR-A* Lo-Mendell-Rubin adjusted likelihood ratio test

|  | **Log likelihood** | **AIC** | **BIC** | **ssaBIC** | **LMR-A** | **Entropy** |
| --- | --- | --- | --- | --- | --- | --- |
| 1 | -17572.97 | 35151.94 | 35168.78 | 35159.25 |  |  |
| 2 | -16489.07 | 32990.13 | 33023.81 | 33004.75 | 2076.87  .000 | .879 |
| 3 | -16169.44 | 32356.89 | 32407.41 | 32378.82 | 612.43  .000 | .857 |
| 4 | -16053.93 | 32131.87 | 32199.23 | 32161.11 | 221.32  .041 | .833 |
| 5 | -15934.57 | 31899.14 | 31983.34 | 31935.69 | 228.71  .011 | .806 |
| 6 | -15874.13 | 31784.27 | 31885.31 | 31828.13 | 115.79  .338 | .801 |
| 7 | -15825.08 | 31692.17 | 31810.05 | 31743.33 | 93.98  .012 | .809 |

**Table S2. Fit Indices for the Latent Class Growth Analysis for Traumatic Stress.**

|  | **Log likelihood** | **AIC** | **BIC** | **ssaBIC** | **LMR-A** | **Entropy** |
| --- | --- | --- | --- | --- | --- | --- |
| 1 | -14416.66 | 28839.33 | 28856.17 | 28846.64 |  |  |
| 2 | -13300.80 | 26613.60 | 26647.28 | 26628.21 | 2138.12  .000 | .91 |
| 3 | -13104.55 | 26227.10 | 26277.62 | 26249.03 | 376.03  .000 | .89 |
| 4 | -12888.56 | 25801.13 | 25868.49 | 25830.36 | 413.85  .005 | .88 |
| 5 | -12769.15 | 25568.31 | 25652.51 | 25604.85 | 228.80  .013 | .86 |
| 6 | -12699.96 | 25435.93 | 25536.97 | 25479.78 | 132.58  .278 | .87 |
| 7 | -12632.17 | 25306.35 | 25424.23 | 25357.51 | 129.89  .666 | .86 |

*AIC* Akaike Information Criterion; *BIC* Bayesian Information Criterion; *ssaBIC* sample size adjusted Bayesian Information Criterion; *LMR-A* Lo-Mendell-Rubin adjusted likelihood ratio test

**Additional references**

Akaike, H. (1987). Factor analysis and AIC. *Psychometrika, 52*(3), 317-332. doi:10.1007/BF02294359

Buhr, K., & Dugas, M. J. (2002). The intolerance of uncertainty scale: Psychometric properties of the English version. *Behaviour Research and Therapy, 40*(8), 931-945.

Hoffman, L. (2015). *Longitudinal analysis: Modeling within-person fluctuation and change*. New York, NY: Routledge.

Hughes, M. E., Waite, L. J., Hawkley, L. C., & Cacioppo, J. T. (2004). A short scale for measuring loneliness in large surveys: Results from two population-based studies. *Research on Aging, 26*(6), 655-672.

Lo, Y., Mendell, N. R., & Rubin, D. B. (2001). Testing the number of components in a normal mixture. *Biometrika, 88*(3), 767-778. doi:10.1093/biomet/88.3.767

Masyn, K. E. (2013). Latent class analysis and finite mixture modeling. In T. D. Little (Ed.), *The Oxford Handbook of Quantitative Methods, Vol 2: Statistical Analysis* (pp. 551-611). New York, NY: Oxford University Press.

Muthén, L. K., & Muthén, B. O. (2018). *Mplus user's guide (8th ed.)*. Los Angeles, CA: Muthen & Muthen.

Nylund, K. L., Asparouhov, T., & Muthén, B. O. (2007). Deciding on the number of classes in latent class analysis and growth mixture modeling: A Monte Carlo simulation study. *Structural Equation Modeling: A Multidisciplinary Journal, 14*(4), 535-569. doi:10.1080/10705510701575396

Sapp, S. G., & Harrod, W. J. (1993). Reliability and validity of a brief version of Levenson's locus of control scale. *Psychological Reports, 72*(2), 539-550.

Schafer, J. L., & Graham, J. W. (2002). Missing data: Our view of the state of the art. *Psychological Methods, 7*(2), 147–177.

Schwarz, G. (1978). Estimating the dimension of a model. *The Annals of Statistics, 6*(2), 461-464.

Sclove, S. L. (1987). Application of model-selection criteria to some problems in multivariate analysis. *Psychometrika, 52*(3), 333-343. doi:10.1007/BF02294360

Smith, B. W., Dalen, J., Wiggins, K., Tooley, E., Christopher, P., & Bernard, J. (2008). The brief resilience scale: Assessing the ability to bounce back. *International Journal of Behavioral Medicine, 15*(3), 194-200.

Tomás-Sábado, J., Gómez-Benito, J., & Limonero, J. (2005). The Death Anxiety Inventory: A revision. *Psychological Reports, 97*, 793-796. doi:10.2466/pr0.97.3.793-796
